# Piezoelectric Active Humidity Sensors Based on Lead-Free NaNbO_3_ Piezoelectric Nanofibers

**DOI:** 10.3390/s16060833

**Published:** 2016-06-07

**Authors:** Li Gu, Di Zhou, Jun Cheng Cao

**Affiliations:** 1 The Key Laboratory of Terahertz Solid-State Technology, Shanghai Institute of Microsystem and Information Technology, Chinese Academy of Science, Shanghai 200050, China; lgu@mail.sim.ac.cn; 2 School of Physics and Information Engineering, Jianghan University, Wuhan 430056, China; zhdijhu@gmail.com

**Keywords:** sodium niobate, piezoelectric energy harvesting, nanofibers, self-powered, humidity sensors

## Abstract

The development of micro-/nano-scaled energy harvesters and the self-powered sensor system has attracted great attention due to the miniaturization and integration of the micro-device. In this work, lead-free NaNbO_3_ piezoelectric nanofibers with a monoclinic perovskite structure were synthesized by the far-field electrospinning method. The flexible active humidity sensors were fabricated by transferring the nanofibers from silicon to a soft polymer substrate. The sensors exhibited outstanding piezoelectric energy-harvesting performance with output voltage up to 2 V during the vibration process. The output voltage generated by the NaNbO_3_ sensors exhibited a negative correlation with the environmental humidity varying from 5% to 80%, where the peak-to-peak value of the output voltage generated by the sensors decreased from 0.40 to 0.07 V. The sensor also exhibited a short response time, good selectively against ethanol steam, and great temperature stability. The piezoelectric active humidity sensing property could be attributed to the increased leakage current in the NaNbO_3_ nanofibers, which was generated due to proton hopping among the H_3_O^+^ groups in the absorbed H_2_O layers under the driving force of the piezoelectric potential.

## 1. Introduction

Nowadays, the development of high-performance humidity sensors has attracted great attention because monitoring and controlling environmental humidity is important for industry, agriculture, and people’s daily lives [1]. Thanks to their high sensitivity, simple fabrication processes and device structures, as well as the high integrity of IC techniques, low-dimensional semiconductor nanomaterials are widely investigated as resistance-type humidity sensing materials [2,3,4,5,6]. Their working mechanism can be attributed to the increase in conductivity after the water molecules are absorbed on the surface of the sensing layers. Moreover, there are also capacitive sensors with a high dielectric response to the variation in relative humidity, which exhibit fast response speed and high temperature compensation [7,8,9]. However, both the resistive and capacitive humidity sensors require an external power unit such as lithium ion batteries. The relatively large size of the batteries and their need for frequent recharging or replacement has limited the miniaturization and intelligentization of modern smart-sensor systems.

Since the power consumption of the micro-scaled sensors has decreased to a much lower level due to the miniaturization of sensing layers, the electrical power generated by energy harvesters such as solar cells, thermoelectric devices, and piezoelectric nanogenerators is sufficient to maintain the sensors [10]. Among them, the piezoelectric nanogenerators that can convert mechanical vibration energy into electrical power has exhibited unique advantages compared to the former versions because mechanical energy is ubiquitous in our environment [11,12,13,14]. As a result, mechanical energy harvesting will be less affected by working conditions than optical and thermal energies. For instance, Xu *et al.* have demonstrated self-powered pH and UV sensors with a ZnO nanowire-based piezoelectric nanogenerator as their powering sources [15]. Partial voltage applied to the sensors by the nanogenerators changed with the sensor resistance when the pH value and UV irradiation around the sensor changed. Moreover, Lee and co-authors have reported a self-powered environment sensor, which consists of a piezoelectric nanogenerator and single-wall carbon nanotubes (SWCNT) [13]. The sensor could be used for detecting mercury ions in a water solution by lighting up a light-emission diode (LED) with the nanogenerators once the concentration of mercury ions is beyond the alarm level. However, the integration of nanogenerator and sensor units in the self-powered system is complicated because the interference of mechanical vibration from the nanogenerators on the sensor units should be eliminated to guarantee system stability.

Recently, a novel type of self-powered sensor system was demonstrated by Zhu and his co-authors, showing that the ZnO nanowires could act as both the piezoelectric energy harvester and the sensor unit [10,16,17]. The working mechanism of such “active sensors” can be attributed to the polarization screening effect, which was induced by the redistribution of free charge carriers in the nanowires under piezoelectric potential. As the free charge carrier density of the ZnO nanowires was sensitive to the surface-absorption of chemicals such as hydrogen, ethanol, water, and glucose, those devices could be used for detecting the target subject by monitoring the variation in generated output voltage [18,19,20,21]. Therefore, both the piezoelectric and chemical sensing properties of the nanowires are crucial for building high-performance active sensors. Although the ZnO nanowires can play both roles in the devices, the limited piezoelectric constant and poor gas sensing selectivity may limit the practical application of such active sensors. As reported, the perovskite NaNbO_3_ nanofibers (NFs) have exhibited ultra-high sensitivity and selectivity to water molecules at room temperature. The surface-absorption of water molecules leads to a great enhancement of a NF’s conductivity [22]. Simultaneously, the NaNbO_3_ nanowires have also been reported to have an outstanding piezoelectric energy-harvesting performance by Jung *et al.* [23]. Those results inspired us to consider the potential of the NaNbO_3_ nanowires for building high-performance active humidity sensors.

In this work, the perovskite NaNbO_3_ NFs were synthesized using a far-field electrospinning method. A flexible active humidity sensor based on the NaNbO_3_ NFs was assembled on the polydimethylsiloxane (PDMS) substrate. The energy-harvesting behavior of the devices under various humidity conditions was investigated. The device exhibited highly sensitive active humidity sensing performance at room temperature. Moreover, the working mechanism of the active humidity sensor was also discussed in detail.

## 2. Materials and Methods

The NaNbO_3_ NFs were firstly synthesized on the clean silicon substrates through a far-field electrospinning method. The detailed experimental procedures are as follows: First, the NaNbO_3_ sol-gel of 0.4 mol/L was prepared by the reflux reaction of the niobium ethoxide and sodium acetate anhydrous in the mixture of acetic acid and 2-methoxyethanol with a volume ratio of 1:3. The reaction lasted for 1 h at 95 °C and was protected in a dry N_2_ atmosphere to prevent hydrolysis of the niobium ethoxide. All chemical reagents were analytically pure and purchased from Alfa Aesar in China. After the reaction, a transparent yellow NaNbO_3_ solution was obtained. Then, the electrospinning precursor was prepared by mixing the NaNbO_3_ solution with the ethanol solution of poly(vinylpyrrolidone) (PVP, MW-1300000, 0.08 g/mL). After stirring for 12 h at room temperature, the precursor was transferred into a 5-mL plastic syringe, which was then positioned onto the syringe pump to electrospin the NFs. The clean silicon substrate was positioned on the metal sample collector, which was placed 10 cm away from the syringe tip. The electrospinning process was then started at a voltage of 1.4 kV/cm, and the precursors ejected at a constant rate of 0.36 mL/h. Finally, the substrates were covered by white composite NFs of the NaNbO_3_ solution, and the PVP was dried at 80 °C for 5 h. The dried composite NFs were firstly pre-sintered at 450 °C for 1 h to combust the carbons and then annealed at 700 °C to form the perovskite NaNbO_3_ polycrystalline NFs. All heat treatments were done in an oxygen atmosphere. The structure and morphology of the samples was characterized by the X-ray diffractometer (XRD, Bruker D8 Advanced, Cu*K*α, λ = 0.15406 nm) and field-emission scanning electron microscopy (FESEM, JSM7100F).

In order to fabricate the flexible nanogenerators, the prepared NaNbO_3_ NFs were transferred from the silicon substrate onto the flexible PDMS substrate. Firstly, a certain amount of PDMS silicone was spin-coated on the samples and then cured at 60 °C to make the NFs stiff on the PDMS polymers. Then, the silicone was carefully lifted off from the substrate to transfer the NFs onto the soft polymer substrate. After that, the Pt interdigital electrodes (IDEs) were deposited on the NFs/PDMS surface by using a shadow mask during the sputtering process. Finally, the devices were completed after wire leading. Then, the NaNbO_3_ NFs were poled with a DC voltage of 20 kV/cm at room temperature. The electrodes of the devices were then shorted for 30 min to remove the stored charges. The piezoelectric energy harvesting and active humidity sensing performance of the devices were measured with a self-designed measuring system as well as a data acquisition (DAQ) card (National Instruments USB-6210), a digital multimeter (Keithley 2000), a charge amplifier (Shenzhen Cheng Tec, Shenzhen, China, CT5002), and the WS-60A gas sensor testing system. Among them, the Keithley 2000 multimeter was used for testing the output voltage generated by the sensors when the experiment was conducted inside an air-dry oven to test the temperature stability. The DAQ USB-6210 was used to test the output voltage at room temperature.

## 3. Results and Discussions

**Figure 1:**
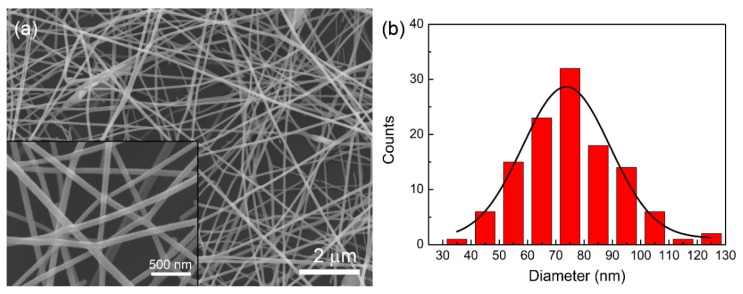
(**a**) The field-emission scanning electron microscopy (FESEM) images of the as-synthesized NaNbO_3_ nanofibers (NFs) after being annealed at 700 °C. The inset picture is the magnified image, and (**b**) is the statistical result of the NFs’ diameters.

**Figure 2:**
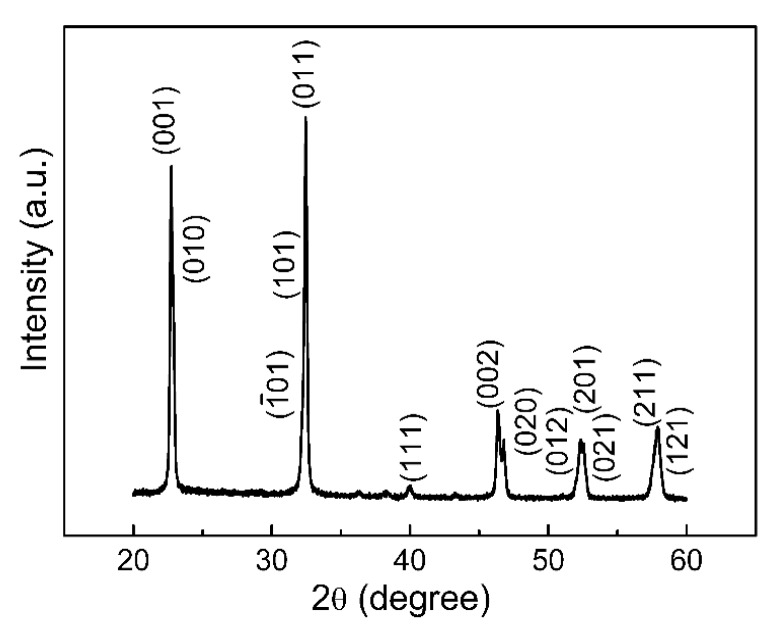
The X-ray diffractometer (XRD) pattern of the NaNbO_3_ NFs after being annealed at 700 °C in oxygen.

Figure 1a shows the SEM image of the as-synthesized NaNbO_3_ NFs on the silicon substrate after the annealing treatment. The NFs were randomly arranged on the substrate with a good size distribution and smooth surface morphology. The average diameter of the NFs was ~78 nm according to the statistical result shown in Figure 1b. The XRD result shown in Figure 2 confirms the perovskite structure of the NFs, with all diffraction peaks indexed into the monoclinic phase of NaNbO_3_ (JCPDS card No. 74-2437).

**Figure 3:**
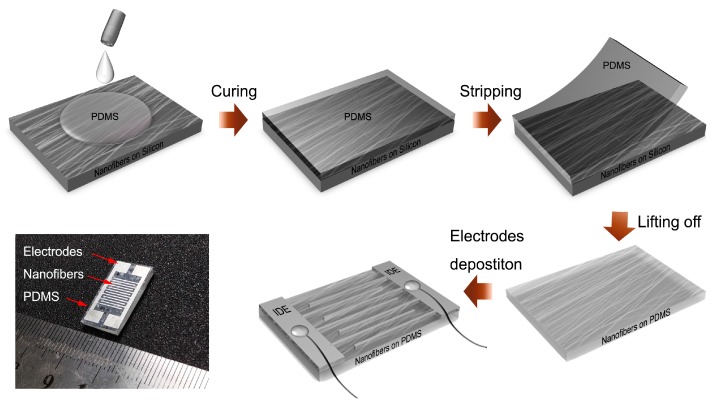
NaNbO_3_ NF-based active sensors fabrication procedure.

Figure 3 shows the schematic fabrication procedure of the flexible NaNbO_3_ active sensor. Because the heat treatment is necessary for the formation of perovskite NaNbO_3_ NFs, silicon substrates were used for the synthesis of the NFs. Due to the porous structure of the NFs on the silicon substrate, the top layer of the NFs could be packaged by the PDMS soft polymers through the spin-coating and the curing process. Thereafter, the NFs could be easily transferred onto the flexible PDMS substrate by lifting off the PDMS layer. After the IDE deposition, the flexible device based on the electrospun NaNbO_3_ NFs could be obtained. As shown by the photo image in Figure 3, the as-fabricated device was approximately 2.0 × 1.0 × 0.2 cm^3^ in dimension.

**Figure 4:**
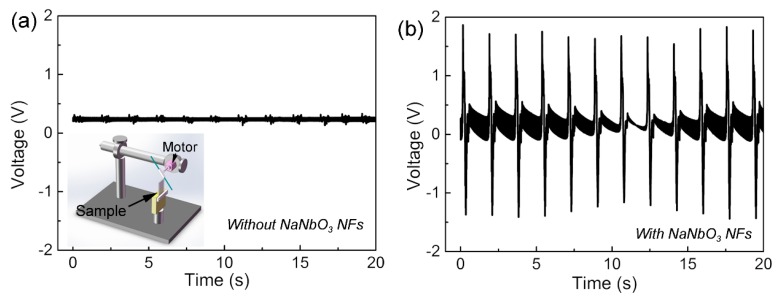
The output voltage generated by the active sensor (**a**) without and (**b**) with the NaNbO_3_ NFs when the sensors were periodically bent by a rotating stick.

The inset picture in Figure 4a illustrates the piezoelectric energy-harvesting performance-testing method, in which the poled device is fixed at one end and forced by a rotating stick to bend. As shown in Figure 4a, the output voltage generated by the device without NaNbO_3_ NFs was lower than 0.2 V, which was too weak to be recognized from the strong background noise, and only 10% of the output voltage was generated by the sensors with NaNbO_3_ NFs (Figure 4b). The much lower voltage signal generated from the blank sample may be due to releasing stored remnant charges between the electrodes, because the thickness of the PDMS layer changed during the bending process. These results suggest the energy-harvesting behavior of the device should be attributed to the piezoelectric effect of the NaNbO_3_ NFs.

**Figure 5:**
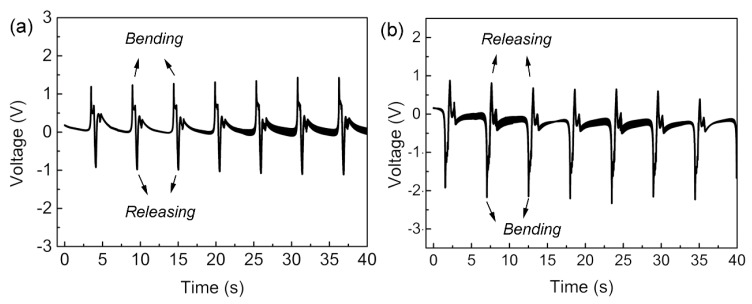
The output voltage generated by the NaNbO_3_ sensor with the electrodes connected (**a**) straight and (**b**) in reverse to the DAQ system.

Figure 5 shows the inversion of voltage polarity when the electrodes of the device were reversely connected to the DAQ card. When it was connected to the DAQ card with forward direction, a positive voltage peak was firstly recorded with the device bending motion (Figure 5a). The positive polarity of the voltage peak can be attributed to the axial stretching of NaNbO_3_ NFs under the bending state, which may generate the piezoelectric potential along the same direction of the poling electrical field. Once the connection was reversed, the device bending resulted in the generation of negative voltage peaks (Figure 5b). Those results also confirmed that the piezoelectric effect of the NaNbO_3_ NFs was the predominant mechanism of the energy-harvesting behavior [24].

**Figure 6:**
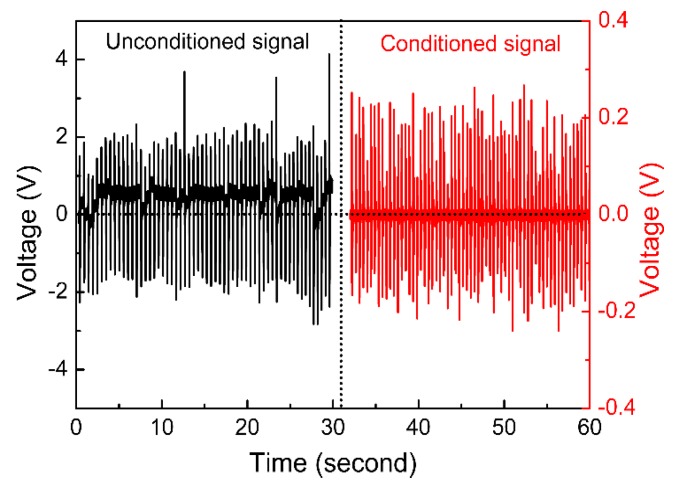
Comparison of recorded voltage signals generated by the NaNbO_3_ sensors with and without signal conditioning.

It is known that the sensitivity of sensor systems mainly depends on the signal-to-noise ratio (SNR) of the signals. Because of the high-frequency sampling and much higher impedance of the NaNbO_3_ NFs (up to TΩ) compared with the input impedance of the DAQ cards (~MΩ), the noise in the recorded voltage signals was unstable and too high to recognize the relatively lower voltages during the sampling process [25]. This limited the sensitivity of the energy harvesters as the piezoelectric active sensors, in which the generated voltage was the sensitivity signal for the detection of humidity changes. As a result, a charge amplifier was used for the signal conditioning, including the impedance matching and filtering. Figure 6 shows the comparison of recorded voltage signals by the DAQ cards between the unconditioned and the conditioned signals. As shown, the ratio between the output voltage and noise amplitude was ~10 for the unconditioned signals and could be increased to ~20 after signal conditioning. Although the voltage amplitude decreased due to the impedance matching, the increased SNR could enhance the sensitivity of the sensors. Moreover, the stability of the voltage signal also improved due to filtering the high-frequency noise, which resulted in a stable base line signal and improved the accuracy of the sensors.

**Figure 7:**
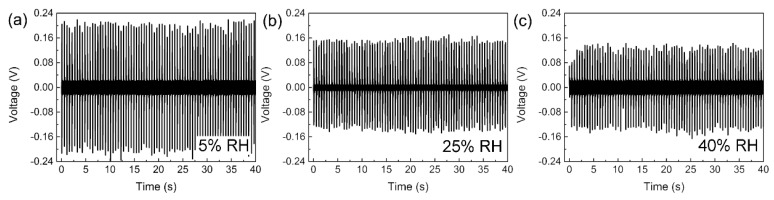

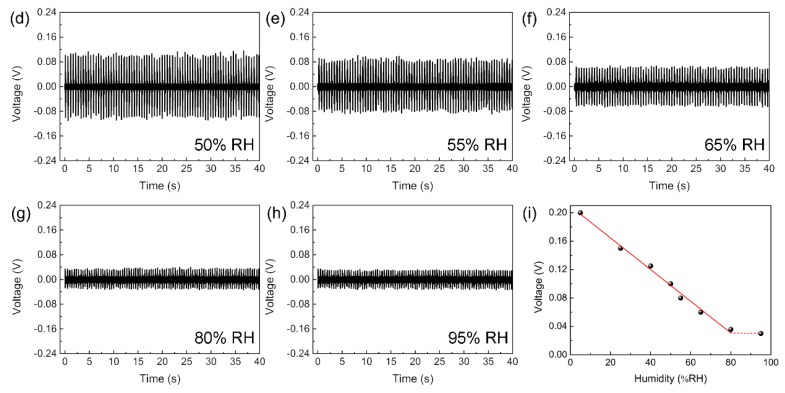
(**a**–**h**) The output voltage generated by the active sensor at different humidity conditions; (**i**) the relationship between the peak value of output voltage and the humidity.

Figure 7 shows the piezoelectric active humidity response of the NaNbO_3_ sensors with the voltage signal conditioned by the charge amplifier. The sensing performance was tested by changing the humidity of the environment when the NaNbO_3_ sensors were continuously vibrating under the knocks of the rotating stick. As shown in Figure 7a–h, the sensor could generate distinguishable impulsive voltage with the humidity changing from 5% to 95% RH, where the peak-to-peak value of the voltage decreased from 0.4 to 0.06 V. Figure 7i shows the relationship between the voltage and the humidity of the environment. The voltage amplitude exhibited a negative linear correlation with the humidity varying from 5% to 80% RH and saturated when the humidity rose higher than 80% RH. As reported, the piezoelectric energy-harvesting behavior of the NaNbO_3_ NFs could be attributed to the electron flow in the external circuit under the driving force of the piezoelectric potential [26,27,28]. The electrons would accumulate at the positive side of the electrode due to the Schottky potential barrier between the Pt electrode and NaNbO_3_ NFs and compensate the piezoelectric potential. Once the piezoelectric potential was removed due to the release of strain, the accumulated electrons would flow back to the positive side along the external circuit and generate a negative voltage peak. As reported, the electrical field applied along the axial direction of the NaNbO_3_ NFs could lead to protons hopping among the surface-absorbed H_3_O^+^ groups [22,29]. Therefore, an additional charge flow could be generated on the surface of the NaNbO_3_ NF when the piezoelectric potential was generated due to bending motion. This additional charge flow would lead to leakage current inside the piezoelectric materials and thus decrease the output voltage of the devices. When the environmental humidity changed, the concentration of protons on the surface of the NFs also changed. As a result, the output voltage changed due to the variation in leakage current. Moreover, the leakage current density in the NFs was in a linear correlation with the concentration of protons and absorbed water molecules [17]. Therefore, the output voltage exhibited a negative linear relationship with the environmental humidity at 5% to 80% RH. However, the absorption of water molecules was saturated when the humidity increased further (>80% RH), which resulted in the slight decrease in output voltage shown in Figure 7i. Finally, the sensitivity of the active sensor could be obtained as ~2 mV/%RH according to the linear result fitting within the humidity range of 5% to 80% RH. The relatively lower sensitivity of this NaNbO_3_ sensor compared to the reported CeO_2_/ZnO and SnO_2_/ZnO sensors should be attributed to the decreased output voltage amplitude after the signal conditioning. However, it is worth noting that the structure of the NaNbO_3_ nanofiber-based sensors was much more stable than the sensors based on the ZnO nanorod arrays, which was assembled through the stacking of the top electrode and the nanorod arrays.

**Figure 8:**
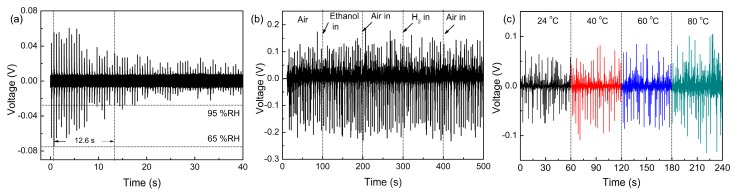
(**a**) The transient response of the sensor when the humidity increased from 65% to 95% RH. (**b**) The transient response of the sensor when ethanol steam and H_2_ was introduced and removed, respectively. (**c**) The output voltage of the sensor at 38% RH under different temperature.

Moreover, the sensor also exhibited fast response speed to the variation in humidity. As shown in Figure 8a, the response time of the sensors for the humidity changing from 65% to 95% RH is ~12 s. Considering the time taken for the change in humidity, the real value of the response time should be shorter. Figure 8b shows the output voltage generated by the sensors when ethanol steam and H_2_ gas was introduced to and then removed from the testing chamber, respectively. The output voltage did not exhibit any change during the introducing and removing process of ethanol and H_2_, which confirmed the excellent selectivity of the sensor. In order to evaluate the influence of temperature on the sensing result, the energy conversion property of the sensors under 38% RH was tested when the temperature increased from 24 to 80 °C. As shown in Figure 8c, no obvious change in the voltage amplitude could be found, which suggested that the sensor possessed great temperature stability during the testing process.

## 4. Conclusions

In this work, monoclinic NaNbO_3_ piezoelectric NFs with a uniform size distribution were synthesized using a far-field electrospinning method. By lifting off the PDMS layer coated on the NFs and the electrode deposition, a flexible active humidity sensor was obtained. The sensors could generate an impulsive output voltage with an amplitude up to 2 V during the vibration process. The energy-harvesting performance was confirmed to have originated from the piezoelectric effect of the NaNbO_3_ NFs. By conditioning the voltage signal with a charge amplifier, the noise in the recorded voltage signals was suppressed by half, which could be attributed to matching impedance and filtering high frequency signals. The output voltage of the NaNbO_3_ sensors after the signal conditioning exhibited a negative linear correlation with the environmental humidity when the humidity changed from 5% to 80% RH. The humidity-dependent output voltage provides the potential of the NaNbO_3_ device to be applied as an active humidity sensor with the sensitivity of 2 mV/%RH. The variation in output voltages with humidity can be attributed to the humidity-dependent leakage current inside the NaNbO_3_ NFs, which was induced by protons hopping among the surface-absorbed H_3_O^+^ under the driving force of piezoelectric potential. The active sensor also exhibited a fast response speed, as well as great selectivity and temperature stability. The active humidity sensor based on NaNbO_3_ NFs provides an effective solution for self-powered sensor systems with high sensitivity, simple structure and fabrication processes, and low production costs.

## Abbreviations

The following abbreviations are used in this manuscript:

SWCNTs	single-wall carbon nanotubes
NFs	Nanofibers
LED	light emission diode
SNR	signal-to-noise ratio
XRD	X-ray diffraction
DAQ	data acquisition
IDE	interdigital electrode
